# CHALLENGES AND BARRIERS FOR SERVICE PROVIDERS IN RURAL SETTINGS: AN ELDER-ABUSE NEEDS ASSESSMENT

**DOI:** 10.1093/geroni/igz038.1776

**Published:** 2019-11-08

**Authors:** Louise Peitz, Jeong Eun Lee, Megan Gilligan, Naomi R Meinertz

**Affiliations:** 1 Iowa State University, Ames, Iowa, United States; 2 Iowa State, Ames, Iowa, United States

## Abstract

There has been concern about the increasing prevalence of elder abuse in the rural settings. This trend has called for more coordinated efforts to address elder abuse in the rural community. The current literature regarding elder abuse suggest there is more to learn about systematic obstacles service providers face. The framework applied in this study include elements of Bronfenbrenner’s Bioecological model, showing a multilevel application to the obstacles agencies encounter when supporting else abuse victims. Little research has been conducted on the providers who work with elders in the rural context. In this study, based on the needs assessment of Iowa, we have examined the perceived barriers service providers face. Our sample included 222 providers across Iowa who took an online survey. The survey was initially sent to members of the Coordinated Community Response team of Iowa. Our findings indicated that service providers revealed multilevel challenges (i.e., individual vs. organization level) in working with elder abuse cases. Service providers’ perceived effectiveness training was negatively associated with the quality of training they evaluated (β=.24, p<.05). The data also showed us that a large portion of agencies offered no training and education on elder abuse. Common themes described by the providers as barriers to addressing abuse was lack of education, social isolation, low awareness and ageism. These results highlight that training for elder abuse in the rural setting needs multilevel systematic efforts.

